# Emergency Department Visits in the United States for Paroxysmal Supraventricular Tachycardia Are Increasing Among Adults: An Analysis from the Nationwide Emergency Department Sample

**DOI:** 10.1016/j.acepjo.2026.100343

**Published:** 2026-03-03

**Authors:** Nihar R. Desai, Charles V. Pollack, Arjun K. Venkatesh, Xiaohui Jiang, Naomi C. Sacks, Anita Holz, David B. Bharucha, Sean D. Pokorney

**Affiliations:** 1 Section of Cardiovascular Medicine, Yale School of Medicine, New Haven, Connecticut, USA; 2 Department of Emergency Medicine, University of Mississippi, Jackson, Mississippi, USA; 3 Department of Emergency Medicine, Yale School of Medicine, New Haven, Connecticut, USA; 4 Yale New Haven Hospital Center for Outcomes Research and Evaluation, New Haven, Connecticut, USA; 5 BlueRidge Life Sciences, Boston, Massachusetts, USA; 6 Milestone Pharmaceuticals, Charlotte, North Carolina, USA; 7 Duke Heart Center, Duke Clinical Research Institute, Duke University, Durham, North Carolina, USA

**Keywords:** adults, emergency department visits, modeling, paroxysmal supraventricular tachycardia, PSVT, atrial fibrillation

## Abstract

**Objectives:**

Paroxysmal supraventricular tachycardia (PSVT) is an arrhythmia that may be challenging to diagnose because of its sudden and episodic nature and its presentation, which may be confused with acute anxiety, especially after spontaneous resolution. Prevalence of PSVT in the United States (US), based on projections to the 2018 US Census, was estimated to be as high as 2.1 million patients with an annual incidence of nearly 300,000. Prior studies report significant rates of health-care resource use among newly diagnosed patients with PSVT, but likely do not reflect contemporary acute care utilization in a population with increased PSVT prevalence. In this study, we quantified annual US emergency department (ED) visits for PSVT and examined temporal trends in PSVT across time among adults aged ≥18 years old.

**Methods:**

We used the Nationwide Emergency Department Sample data to identify adults who visited the ED with a primary diagnosis of PSVT (International Classification of Diseases, Tenth Revision, Clinical Modification [ICD-10-CM]: I47.1) from 2016-2019. We used Agency for Healthcare Research and Quality–provided sampling weights to estimate annual ED visit counts in the US overall and by the presence or absence of comorbid atrial fibrillation (AFib)/atrial flutter (AFl). We examined demographic characteristics, calculated annual visit rates per 10,000 US population, and assessed visit trends 2016-2019. Sensitivity analyses were conducted in which ED visits were defined primarily for PSVT when PSVT was the primary diagnosis and the ED visit resulted in hospitalization or when PSVT was the diagnosis in any position, and the visit resulted in discharge. We used Poisson regression, based on 2016-2019 visit increases, to project estimates of PSVT visits through 2030.

**Results:**

ED visits for PSVT increased 8.3% over the study period (from 129,219 to 139,992; *P* < .0001); ED visit rates per 10,000 also increased, from 5.16 ( 95% CI, 4.87-5.45) to 5.46 ( 95% CI, 5.18-5.73; *P* = .0029). ED visits for PSVT as a proportion of all ED visits also increased significantly (*P* < .0001). The majority of ED visits in 2019, the final study year (N = 139,992; 95% CI, 132,913-147,070), were for females (59.3%) and individuals aged <65 years (62.6%). Most visits involved patients without secondary diagnoses of AFib/AFl (86.0%; 120,377/139,992); 24.2% resulted in inpatient hospitalizations, and 10.1% incurred observation stays. Sensitivity analyses, including diagnosis in any position, identified an additional 119,914 visits in 2019, for a total of 259,916 ED visits for PSVT, with similar characteristics, but larger proportions of observation stays (19.9%). Based on the rate of change from 2016-2019, annual US ED visits with a primary diagnosis of PSVT are projected to reach nearly 180,000 among adults by 2030.

**Conclusion:**

ED visits due to PSVT in the US have increased significantly among adults. Outpatient interventions for PSVT that reduce the need for an ED visit may minimize the economic burden of PSVT for patients, providers, and payers.

**The Bottom Line**

From 2016-2019, emergency department (ED) visits coded for paroxysmal supraventricular tachycardia (PSVT) increased 8% from 129,000 to 140,000, with most patients under 65 years old and the majority female. Including all diagnosis positions, total visits in 2019 reached nearly 260,000. Nearly 1 in 4 visits led to hospitalization, and 1 in 10 visits led to observation stays. If the current trends continue, visits for PSVT could exceed 180,000 annually by 2030. These findings suggest that better outpatient management, earlier diagnosis, and patient education could reduce emergency visits and lessen the overall health care burden.

## 1 Introduction

### 1.1 Background

Paroxysmal supraventricular tachycardia (PSVT), a cardiac arrhythmia characterized by sudden episodic tachycardia,^1^^,^^2^ is the second most common tachyarrhythmia, after atrial fibrillation (AFib). PSVT symptoms include palpitations, weakness or fatigue, dizziness, fainting, chest pain, and a regular but racing heartbeat of 100 to >250 beats per minute that can start and stop abruptly.^1^^,^^2^ Although confirmatory diagnosis is well standardized using a 12-lead electrocardiogram (ECG) or ambulatory monitoring^1^^,^^2^ during an episode, PSVT can be difficult to diagnose because of its abrupt onset and termination, as well as its episodic nature.^1^ Previous studies reported heterogeneity in diagnostic coding, with PSVT misdiagnosed as other arrhythmias or noncardiac diagnoses such as acute anxiety, thereby limiting the understanding of PSVT burden in the general population.^3^^,^^4^ Episode frequency and severity can also vary across patients. A recent study estimates a PSVT incidence of nearly 300,000 annually and a prevalence of 2.1 million in the US, of which approximately 1.3 million are estimated to have PSVT without comorbid AFib or atrial flutter (AFl).^5^

PSVT is commonly managed in the ED with treatments varying based on the type of rhythm observed on the ECG and the individual’s hemodynamic stability.^1^ Currently available noninvasive PSVT treatment options for acute PSVT episodes remain limited, primarily consisting of oral pharmacotherapy and vagal maneuvers. The “pill-in-the-pocket” strategy, involving intermittent administration of oral β-blockers or calcium channel blockers, offers a potential approach but is supported by limited published evidence, and is associated with variable efficacy with slow time-to-effect.^6^^,^^7^ Several recent studies have found significant increases in health care resource utilization and costs in the years following PSVT diagnosis, reflecting substantially more visits to the ED and hospitalizations following diagnosis of PSVT, as well as higher expenditures for patients with commercial and Medicare coverage compared with matched controls who did not have PSVT.^8^, ^9^, ^10^ These higher annual rates of ED visits, hospitalizations, physician office visits, and diagnostic testing among patients with PSVT compared with matched controls without PSVT were observed to continue in the first 3 years following diagnosis.^8^^,^^11^

### 1.2 Importance

Estimating the burden of acute PSVT and associated health care resource use has proven to be challenging, but it is necessary to inform clinical practice, guide resource allocation, and identify opportunities for improving patient care. Prior studies that highlighted the burden of PSVT, including a study of ED visits for PSVT from 1993 to 2003, have relied on the International Classification of Diseases, Ninth Revision (ICD-9-CM) PSVT diagnosis code, which is less comprehensive than the current ICD-10 PSVT diagnosis code introduced October 1, 2015.^8^ Further, prior studies conducted in the ICD-9 coding era demonstrated that a wide range of up to 8 diagnosis codes were used in the primary diagnosis coding position for encounters found to have PSVT arrhythmias confirmed by adjudication.^3^^,^^4^ One study showed that only 39% of incident PSVT originated from the PSVT ICD-9-CM 427.0 diagnosis code and that 91% of PSVT cases could be identified using 4 ICD-9 diagnosis codes (426.7, 427.0, 427.89, and 427.9).^4^ Given the heterogeneity in PSVT coding practices, characterizing PSVT at a national level, including assessing trends in PSVT and PSVT-related health care resource use, has been challenging. The more comprehensive ICD-10 diagnosis code may better address these challenges.

### 1.3 Goal of This Investigation

The goal of the present study was to characterize annual ED visits for PSVT among United States (US) adults in 2019 and examine trends in ED visits in the years following the introduction of the more comprehensive ICD-10 PSVT diagnosis code (2016-2019).

## 2 Methods

### 2.1 Study Design and Data Source

In this serial cross-sectional analysis, we examined the annual number of US ED visits for PSVT in the adult (aged ≥18 years) population. Our data source was the Agency for Healthcare Research and Quality Healthcare Cost and Utilization Project (HCUP)’s Nationwide Emergency Department Sample (NEDS) data set,^12^ which constitutes the largest, all-payer, nationally representative collection of ED care in the US, covering 993 hospitals across 39 states and the District of Columbia. The NEDS data comprises a 20% stratified sample of ED visits and, unweighted, captures approximately 30 million ED visits in each year of the database. National estimates can be calculated using the sampling weights that are provided by the HCUP; when this weighting is used, 127 million ED discharges are represented in NEDS. The complex sampling design was accounted for by incorporating NEDS stratum, hospital cluster, and discharge weights to generate nationally representative estimates of ED visits. This study examined ED visits between 2016 and 2019, as the ICD-10-CM codes were implemented in October 2015, and these are the most recent data years unaffected by well-documented and transient impacts of the COVID-19 pandemic on acute care use. The study focuses on PSVT ED visits in 2019 using the most recent pre-COVID-19 data and examines trends in ED visits from 2016-2019.

### 2.2 Study Measures

Our primary study measure was ED visits for PSVT, identified in the NEDS as ED visits with a PSVT diagnosis (ICD-10-CM code I47.1) in each year, 2016-2019. We focused on ED visits among adults aged ≥18 years. We limited our initial analysis to ED visits with a PSVT diagnosis code in the primary position, as ED visits that result in hospital admission are billed and paid as part of the admission, with reimbursement based upon the first-position diagnosis, which is considered the chief reason for an admission. In sensitivity analyses, we identified PSVT ED visits as those with a primary diagnosis of PSVT that resulted in an inpatient admission or a diagnosis of PSVT in any position where the patient was discharged. This definition reflects known differences in coding requirements and patterns between the inpatient and outpatient hospital claims files assembled by HCUP in NEDS. To implement it, we added ED visits with a secondary diagnosis of PSVT where the patient was discharged to those in the main analysis of ED visits with a primary diagnosis of PSVT, regardless of the visit outcome.

Our measures also included PSVT ED visits with observation stays and those with and without comorbid AFib/AFl. Observation stays were identified by the existence of any of the Current Procedural Terminology codes G0378 (Hospital observation service, per hour) and G0379 (Direct referral of patient for hospital observation care) or by codes that refer to observation care for patients who are admitted to an observation unit for monitoring (99217-99220; 99224-99226; and 99234-99236). ED visits with comorbid AFib/AFl were those with a diagnosis for AFib or AFl (ICD-10-CM codes: I48.0; I48.1x, I48.2x, I48.3, I48.4, I48.9x) (Table S1) in any position other than the position of the PSVT diagnosis.

To evaluate the effect of demographic changes on ED visits for PSVT, including increases in adults aged ≥ 65 years, we examined the proportion of ED visits for PSVT in each study year. We also measured rates of ED visits per 10,000 US population, both overall and by age group (age 18-64 and ≥65 years). We used adult (aged ≥18 years) US population counts from the Surveillance, Epidemiology, and End Results (SEER) Program (SEER∗Stat Software, Version 8.4.3) as the denominator,^13^ and PSVT ED visit counts as the numerator to estimate PSVT ED visit rates per 10,000 US population for each study year.

### 2.3 Study Outcomes

Our study outcomes were weighted numbers of annual PSVT ED visits in the US, overall, and as a proportion of all weighted ED visits. Rates of PSVT ED visits per 10,000 US population, ED visit characteristics, and trends in ED visit numbers, rates, and characteristics over the study period were also outcomes. Visit characteristics were demographics (mean age and age distribution; distribution of visits by gender and by race/ethnicity), distribution of ED visits by expected primary payer (private insurance, Medicare, Medicaid, and unknown), distribution by census region in which the hospital was located, and the proportions of PSVT ED visits that resulted in admission to the same hospital or included an observation stay. Trends in ED PSVT visit rates per 10,000 US population were assessed to determine whether changes in ED visit numbers over the study period reflected changes in the US population, including increases in those aged ≥65 years (also known as “baby boomers”). Unadjusted Poisson regression was conducted to estimate the projected number of ED visits for PSVT from 2020 to 2030 using the rates of change in PSVT ED visits from 2016-2019, which were applied to the US population estimates based on the US Census Projections Tables.^14^

The weighted numbers of ED visits for PSVT are presented as counts (95% CI) and percentages of total ED visits in each year. Continuous variables are presented as means (95% CI) and categoric variables as counts and percentages. Pearson chi-squared tests were used to compare categorical variables and *t*-tests to compare continuous variables among groups; a *P*-value of 0.05 was considered statistically significant.

This study was performed in accordance with HCUP’s Data Use Agreement guidelines. Patient-level records or patient identifiers are not contained in the NEDS data. Analysis of these data are exempt from federal regulations for the protection of human-research participants. As all the data are deidentified, an institutional review board’s approval was not sought for the study's conduct.

All analyses were performed in SAS Version 9.4 of the SAS System for Windows (SAS Institute), with statistical procedures that incorporated weighting to account for the structure of the sample survey data. Per HCUP’s Data Use Agreement, all cell counts ≤ 10 were suppressed and not presented. Graphical visualizations were conducted using GraphPad Prism 9.

## 3 Results

### 3.1 Main Analysis: ED Visits With PSVT as Primary Diagnosis

In 2019, the most recent year unaffected by the COVID-19 pandemic for which study data were available, there were 139,992 (95% CI, 132,913-147,070) ED visits with a primary diagnosis of PSVT, representing 0.12% of all ED visits among adults (aged ≥18 years) in that year (N = 117,352,776) (Table 1). The ED visit rate was 5.46 (95% CI, 5.18-5.73) per 10,000 population. The majority of these PSVT ED visits were for adults aged <65 years (62.6%), with a mean age of 57.8 years; most were female (59.3%) and White (68.5%). Private insurance was the primary payer for more visits (39.4%) than Medicare (37.9%), Medicaid (12.4%), and other/unknown (10.4%).^15^ The South census region, which accounts for 38% of the US population, contained a plurality of visits (37.1%). Approximately 10.1% of ED visits resulted in an observation stay, and 24.2% resulted in admission to the same hospital (Table 1).

**Table 1:** ED visits among adults aged ≥18 years in the United States with PSVT as primary diagnosis with and without atrial fibrillation (AFib)/atrial flutter (AFl), Nationwide Emergency Department Sample (NEDS), 2019

Parameters	Overall	Without AFib/AFl	With AFib/AFl
Total ED visits	117,352,776	N/A	N/A
No. of PSVT ED visits (95% CI), % of total ED visits	139,992 (132,913-147,070), 0.119	120,377 (114,389-126,364), 0.103	19,615 (18,255-20,975), 0.017
Rate (95% CI)^a^	5.46 (5.18-5.73)	4.69 (4.46-4.93)	0.76 (0.71-0.82)
Admitted to the same hospital as ED, N (%)	33,835 (24.2)	22,622 (18.8)	11,213 (57.2)
Observation stays, N (%)	14,113 (10.1)	12,032 (10.0)	2081 (10.6)
Age, mean (95% CI)	57.8 (57.4-58.1)	56.2 (55.9-56.5)	67.4 (66.9- 67.9)
Age group, N (%)			
18-64 y	87,686 (62.6)	80,173 (66.6)	7513 (38.3)
≥65 y	52,305 (37.4)	40,203 (33.4)	12,102 (61.7)
Gender, N (%)			
Male	56,948 (40.7)	47,306 (39.3)	9642 (49.2)
Female	83,035 (59.3)	73,062 (60.7)	9973 (50.8)
Race/ethnicity, N (%)			
White	95,833 (68.5)	81,687 (67.9)	14,145 (72.1)
Black	21,284 (15.2)	18,263 (15.2)	3022 (15.4)
Hispanic	12,372 (8.8)	10,968 (9.1)	1405 (7.2)
Asian or Pacific Islander	3757 (2.7)	3445 (2.9)	313 (1.6)
Native American	570 (0.4)	478 (0.4)	93 (0.5)
Other/unknown^b^	6175 (4.4)	5537 (4.6)	638 (3.3)
Payer, N (%)			
Medicare	53,028 (37.9)	40,804 (33.9)	12,224 (62.3)
Medicaid	17,349 (12.4)	15,711 (13.1)	1638 (8.4)
Private insurance	55,110 (39.4)	50,473 (41.9)	4637 (23.6)
Other/unknown^c^	14,505 (10.4)	13,389 (11.1)	1116 (5.7)
Region of hospital, N (%)			
Northeast	24,120 (17.2)	20,570 (17.1)	3549 (18.1)
Midwest	33,417 (23.9)	28,713 (23.9)	4704 (24.0)
South	51,913 (37.1)	44,344 (36.8)	7569 (38.6)
West	30,542 (21.8)	26,749 (22.2)	3793 (19.3)

ED, emergency department; N/A, not applicable; PSVT, paroxysmal supraventricular tachycardia.

a Rates per 10,000 calculated using the US population counts from 2016-2019 as the denominator.

b Other race/ethnicity includes other, missing, or invalid.

c Other insurance includes self-pay, no charge, missing, or invalid.

The majority of ED visits with PSVT as the primary diagnosis did not have a secondary diagnosis of AFib or AFl (86.0%; 120,377/139,992); and among this cohort, the ED visit rate was 4.69 (95% CI, 4.46-4.93) per 10,000 population (Table 1). By contrast, the ED visit rate for PSVT with AFib or AFl was approximately 6-fold less: 0.76 (95% CI, 0.71-0.82) per 10,000 population. PSVT ED visits with AFib or AFl occurred among older patients (mean age, 67.4 vs no AFib or AFl; 56.2 years), fewer females (50.8% vs 60.7%), more covered by Medicare (62.3% vs 33.9%), and fewer covered by Medicaid (8.4% vs 13.3%) or private insurance (23.6% vs 41.9%). The proportion of ED visits resulting in an observation stay was similar in both groups (10.0% without AFib/AFl vs 10.6% with AFib/AFl); however, a substantially greater proportion of patients with AFib or AFl with a primary diagnosis of PSVT had ED visits resulting in hospital admission compared with those without AFib or AFl (57.2% vs 18.8%). Characteristics of race/ethnicity and census regions were similar between the groups (Table 1).

### 3.2 PSVT ED Visit Trends

**Figure 1:**
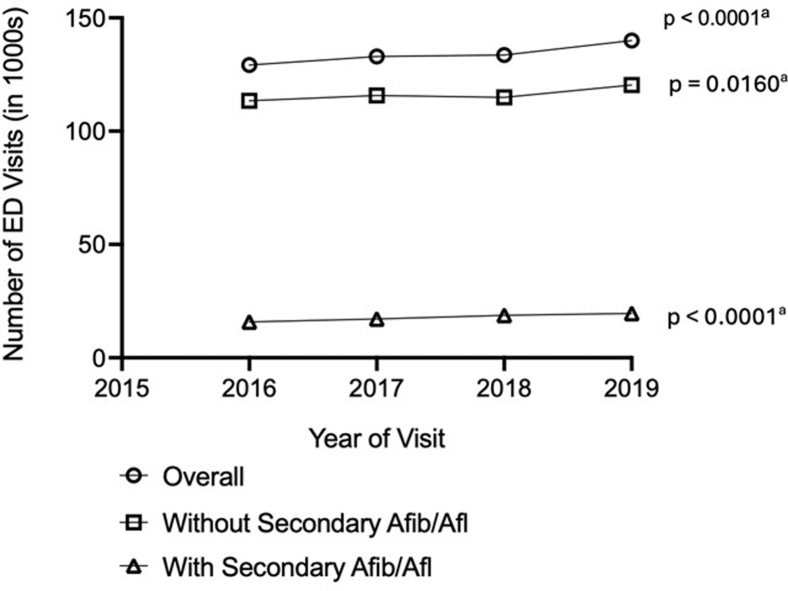
Trends in ED visits among adults aged ≥18 years in the United States with PSVT as primary diagnosis, Nationwide Emergency Department Sample 2016-2019. AFib, atrial fibrillation; AFl, atrial flutter; ED, emergency department; PSVT, paroxysmal supraventricular tachycardia. ^a^ Poisson regression for 2016-2019.

ED visits with PSVT as the primary diagnosis increased significantly, by 8.3%, from 2016-2019 (from 129,219 to 139,992; *P* < .0001) (Fig 1; Table S2). PSVT ED visits as a proportion of all ED visits also increased, from 0.111% of all ED visits in 2016 to 0.119% of all ED visits in 2019 (*P* < .0001). ED visit rates per 10,000 population increased significantly as well, from 5.16 (95% CI, 4.87-5.45) in 2016 to 5.46 (95% CI, 5.18-5.73) in 2019 (*P* = .0029). The proportion of ED visits with an observation stay was greater in 2019 compared with 2016 (10.1% vs 7.6%); the proportion of ED visits that resulted in admission was slightly higher in 2019 compared with 2016 (24.2% vs 23.8%) (Table S2).

PSVT ED visits without a secondary diagnosis of AFib or AFl increased 6.2% from 2016-2019 (from 113,402 to 120,377; *P* = .0160). These ED visits also increased significantly as a proportion of all ED visits (from 0.098% in 2016 to 0.103% in 2019; *P* = .0160). The rate of increase per 10,000 population was lower than overall, growing 3.5% from 4.53 (95% CI, 4.28-4.78) in 2016 to 4.69 (95% CI, 4.46-4.93) in 2019 (*P* = .2154). PSVT ED visits with a secondary diagnosis of AFib or AFl, which were a considerably smaller proportion of all PSVT ED visits, increased 24.0% from 2016-2019 (from 15,817 to 19,615; *P* < .0001) and also increased significantly as a proportion of all ED visits (from 0.014% in 2016 to 0.017% in 2019; *P* < .0001).

PSVT ED visit rates with a secondary diagnosis of AFib or AFl increased significantly (17.1%), from 0.63 (95% CI, 0.58-0.68) in 2016 to 0.76 (95% CI, 0.71-0.82) in 2019 (*P* < .0001). PSVT ED visit rate increases from 2016-2019 were observed in both younger (aged <65 years) and older (aged ≥65 years) patients, including those without and with AFib or AFl. Demographic characteristics remained similar throughout the study period. Consistent with the age distribution, private insurance remained the expected primary payer for more ED visits without AFib/AFl, and Medicare was the expected primary payer for more visits with AFib/AFl (Fig 1; Tables S3 and S4).

### 3.3 Sensitivity Analysis: ED Visits with Primary PSVT Diagnosis and Admissions or Any Position PSVT Diagnosis and Discharge

In a sensitivity analysis where PSVT ED visits were defined as those with a primary position PSVT diagnosis that resulted in admission, as well as those with any position PSVT diagnosis that resulted in discharge, there were a total of 259,916 (95% CI, 243,277-276,554) PSVT ED visits in 2019. These estimated 259,916 ED visits were a subset of all ED visits with a PSVT diagnosis in any position, regardless of the outcome (N, 526,446; 95% CI, 494,156-558,736; Table S5). They included 139,992 with PSVT as the primary diagnosis, identified in our primary analysis, and an additional 119,924 (95% CI, 108,462-131,385) ED visits where the patient was discharged and a PSVT diagnosis was recorded in a secondary position. They represented 0.22% (N = 259,916/117,352,776) of all ED visits among adults aged ≥18 years in 2019 (Table 2).

**Table 2:** ED visits among adults aged ≥18 years in the United States with primary PSVT admitted plus any position PSVT treated and released, Nationwide Emergency Department Sample, 2019

Parameters	Overall	Without AFib/AFl	With AFib/AFl
Total ED visits	117,352,776	N/A	N/A
No. of PSVT ED visits (95% CI), % of total ED visits	259,916 (243,277-276,554), 0.221	216,724 (203,158-230,291), 0.185	43,191 (39,822-46,560), 0.037
Rate (95% CI)^a^	10.13 (9.48-10.78)	8.45 (7.92-8.98)	1.68 (1.55-1.81)
Admitted to the same hospital as ED, N (%)	33,835 (13.0)	22,622 (10.4)	11,213 (26.0)
Observation stays, N (%)	45,075 (19.9)	34,683 (17.9)	10,392 (32.5)
Age, y, mean (95% CI)	58.0 (57.6-58.4)	56.1 (55.7-56.5)	67.6 (67.1-68.1)
Age group, N (%)			
18-64 y	157,785 (60.7)	141,458 (65.3)	16,327 (37.8)
≥65	102,130 (39.3)	75,266 (34.7)	26,864 (62.2)
Gender, N (%)			
Male	98,623 (37.9)	79,017 (36.5)	19,606 (45.4)
Female	161,258 (62.0)	137,673 (63.5)	23,585 (54.6)
Race/ethnicity, N (%)			
White	182,419 (70.2)	150,079 (69.2)	32,341 (74.9)
Black	40,624 (15.6)	34,453 (15.9)	6171 (14.3)
Hispanic	20,046 (7.7)	17,521 (8.1)	2524 (5.8)
Asian or Pacific Islander	6062 (2.3)	5290 (2.4)	772 (1.8)
Native American	1098 (0.4)	914 (0.4)	183 (0.4)
Other/unknown^b^	9667 (3.7)	8467 (3.9)	1200 (2.8)
Payer, N (%)			
Medicare	106,844 (41.1)	79,913 (36.9)	26,931 (62.4)
Medicaid	36,187 (13.9)	32,472 (15.0)	3715 (8.6)
Private insurance	91,900 (35.4)	81,615 (37.7)	10,285 (23.8)
Other/Unknown^c^	24,985 (9.6)	22,724 (10.5)	2261 (5.2)
Region of hospital, N (%)			
Northeast	38,123 (14.7)	32,450 (15.0)	5673 (13.1)
Midwest	69,341 (26.7)	57,072 (26.3)	12,270 (28.4)
South	94,332 (36.3)	78,694 (36.3)	15,637 (36.2)
West	58,120 (22.4)	48,509 (22.4)	9611 (22.3)

AFib, atrial fibrillation; AFl, atrial flutter; ED, emergency department; N/A, not applicable; PSVT, paroxysmal supraventricular tachycardia.

a Rates per 10,000 calculated using the US population counts from 2016-2019 as the denominator.

b Other race/ethnicity includes other, missing, or invalid.

c Other insurance includes self-pay, no charge, missing, or invalid.

Using this expanded definition, rates of ED visits for PSVT were 10.13 (95% CI, 9.48-10.78) per 10,000 US population in 2019. Patient demographic characteristics were similar to those identified using the primary definition, with most visits for individuals aged <65 years (60.7%) with a mean age of 58.0 years (95% CI, 57.6-58.4); a majority of visits were for females (62.0%), most were for individuals identified as White (70.2%), and a plurality of visits (36.3%) were in the South census region. Payer distribution was also similar, although Medicare was the primary payer for more visits (40.1%), compared with private insurance (35.9%), Medicaid (13.7%), and other/unknown (9.6%). Far fewer (13.0%) resulted in inpatient admission, but a substantially larger proportion of visits, 19.9%, resulted in an observation stay, potentially reflecting the expanded definition, with many more ED visits for PSVT that resulted in discharge (Table 2).

The large majority (216,724/259,916; 83.5%) of ED visits identified using this expanded definition did not include a diagnosis of AFib or AFl. The ED visit rate per 10,000 population for PSVT without AFib or AFl was 8.45 (95% CI, 7.93-8.98), considerably higher than the rate for PSVT visits with AFib or AFl (1.68; 95% CI, 1.55-1.81). The characteristics of PSVT ED visits with and without comorbid AFib/AFl, when identified using the expanded definition, were consistent with characteristics of PSVT ED visits with and without AFib/AFl identified using the primary diagnosis of PSVT only. ED visits with comorbid AFib/AFl were for older patients (mean age: 67.6 vs no AFib or AFl: 56.1 years); fewer were for females (52.5% vs no AFib/AFl: 63.5%); more were covered by Medicare (62.4% vs no AFib/AFl: 36.9%); and more were for patients with race/ethnicity recorded as White (74.6% vs no AFib/AFl: 69.2%). More ED visits with comorbid AFib/AFl resulted in hospitalization, compared with those without AFib/AFl (26.0% vs 13.3%), and a substantially larger proportion included an observation stay (32.5% vs 17.9%). As with all ED visits identified using the expanded definition, inpatient admission rates with and without comorbid AFib/AFl were considerably lower and observation rates considerably higher compared with those observed using the primary diagnosis only (Table 2).

### 3.4 ED Visit Trends: Expanded Definition

**Figure 2:**
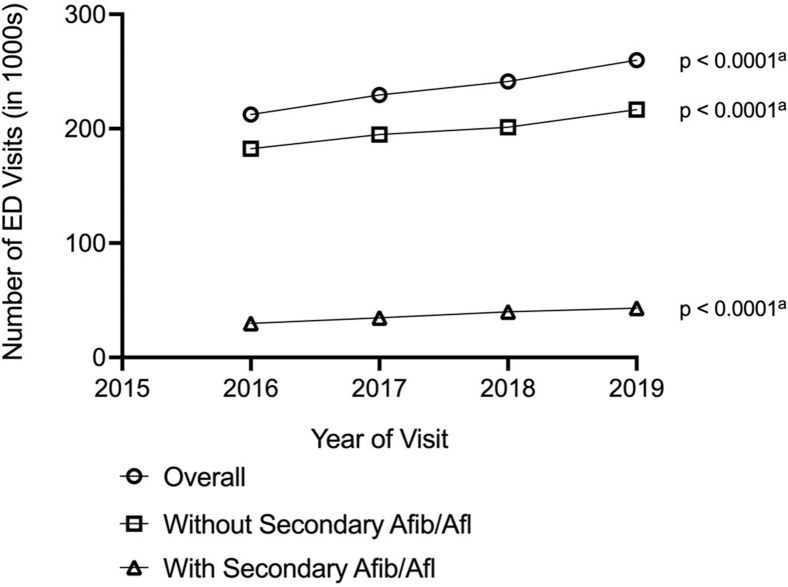
Trends in ED visits among adults aged ≥18 years in the United States with admitted Dx1 PSVT and any position discharged PSVT, Nationwide Emergency Department Sample 2016-2019. AFib, atrial fibrillation; AFl, atrial flutter; ED, emergency department; Dx1, primary diagnosis; PSVT, paroxysmal supraventricular tachycardia. ^a^ Poisson regression for 2016-2019.

Using the expanded definition, PSVT ED visits increased 22.4% over the study period (from 212,374 in 2016 to 259,916 in 2019; *P* < .0001), a more rapid increase compared with PSVT ED visits defined using only the primary diagnosis. The proportion of all ED visits that were for PSVT also increased significantly, both overall and for ED visits with and without secondary AFib or AFl (all *P* < .0001). ED visit rates per 10,000 population also increased more rapidly, using this expanded definition, from 8.48 (95% CI, 7.94-9.03) in 2016 to 10.13 (95% CI, 9.48-10.78) in 2019 (*P* < .0001), with statistically significant increases for the <65 and ≥65 years age groups as well (Fig 2; Table S6). Compared with the more restricted definition, these increases were considerably greater for ED visits overall and for younger (aged 65 years) and older (aged ≥65 years) individuals when stratified by the presence of comorbid AFib/AFl and were statistically significant overall (*P* < .0001 in all cases; Fig 2; Tables S7 and S8). Demographic characteristics and expected primary payers remained similar across all study years.

### 3.5 Model Estimates of ED Visits for PSVT from 2020 to 2030

**Figure 3:**
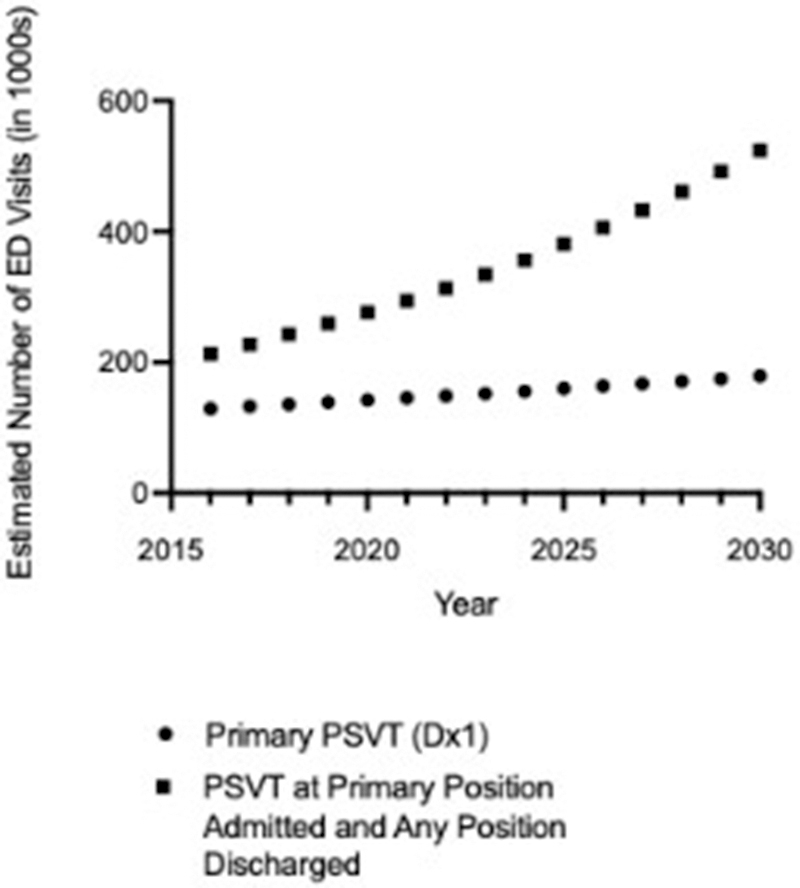
Projected Estimates of ED visits among adults aged ≥18 years in the United States with PSVT in the primary position, and admitted PSVT in the primary position with discharged PSVT in any position, Nationwide Emergency Department Sample (NEDS), 2016-2030. ED, emergency department; Dx1, primary diagnosis; PSVT, paroxysmal supraventricular tachycardia. ^a^ The 2016-2021 US population is from SEER∗Stat; the 2022-2030 US population is interpolated using 2023 National Population Projections Tables.

Based on the trends in ED visits from 2016-2019, we estimated ED visits for PSVT as the primary diagnosis to be 179,000 by 2030, assuming these trends continue. Projections of ED visit numbers were much greater using the expanded definition, reflecting higher rates of increase from 2016-2019, with estimates of 524,160 PSVT ED visits by 2030 (Fig 3; Table S9).

## 4 Limitations

This study has several limitations. The major limitation is its reliance on diagnoses in an administrative database, which does not include physiological measures or medical test results. Therefore, it is possible that some patients were misdiagnosed with PSVT, leading to potential overestimates. However, it is also possible that PSVT episodes had resolved for some patients upon presentation to the ED, resulting in underdiagnosis. Additionally, if some were treated for PSVT and subsequently admitted to the hospital with a different primary diagnosis, our results would underestimate ED visits for PSVT. Further, because the unit of analysis in our study is the ED visit and not each unique patient, we cannot know how many patients visited the ED multiple times. Finally, our projected estimates of ED visit increases are based on a limited set of data points before the COVID-19 pandemic, with the projected estimates highly uncertain.

## 5 Discussion

In this study of comprehensive, nationally representative ED visit data in the US, we estimated an annual prevalence of 140,000 ED visits with a primary diagnosis of PSVT. One in 10 of these visits resulted in an observation stay, and nearly 1 in 4 resulted in an inpatient admission to the same hospital. ED visits for PSVT increased significantly, by 8.3%, from 2016, the first full year with ICD-10-CM coding, through 2019. Additionally, the proportion of ED visits attributable to PSVT significantly increased over the study period. This rise was not statistically significant for PSVT ED visits without secondary AFib or AFl when analyzed using the restricted ED visit definition; however, it achieved significance under the expanded definition. PSVT ED visit rates per 10,000 population also increased significantly over the study period across all age groups, suggesting that these increases did not reflect changes in population demographics. Consistent with prior studies of persons with PSVT, a majority of ED visits and resultant hospitalizations were for those aged <65 years, female, and without a secondary diagnosis of AFib or AFl.

Due to the abrupt onset and termination of PSVT, it is possible that an episode that might lead to transit to or treatment in an ED is often resolved by the time the patient is evaluated. Consequently, given this challenge in PSVT diagnosis, the “true” number of ED visits for PSVT may be > 140,000 estimated for ED visits with a primary PSVT diagnosis. Additionally, when an ED visit does not result in admission, reimbursement does not rely on diagnosis or diagnosis position. Thus, when we expanded the definition of PSVT ED visits to include all visits with a PSVT diagnosis in any position that resulted in discharge, we identified approximately 260,000 ED visits for PSVT in 2019. The demographic characteristics associated with these ED visits were similar to those observed using the more limited definition, as was the proportion of these visits with a comorbid AFib or AFl diagnosis; notably, however, a substantially larger proportion led to an observation stay. Additionally, increases in both the numbers and rates of PSVT ED visits were much greater compared with the initial analysis of ED visits with a primary PSVT diagnosis. ED visits for PSVT may reflect limited evidence-based outpatient treatment options. The increasing number of ED visits may reflect changes in diagnostic coding practice and heightened awareness due to wearable technology and education initiatives in the absence of effective outpatient management options. Based on the clearly upward trends observed from 2016-2019, we estimated that the annual number of ED visits for PSVT as a primary diagnosis will increase to ∼180,000 by 2030, with a potentially larger increase using the broader definition that includes all ED visits for which the patient is discharged and there is a PSVT diagnosis in any position. ED visits for PSVT may be mitigated through improved outpatient management, earlier diagnosis, and patient education, thereby reducing the health care burden and improving the quality of life for patients with PSVT. Clinical trial results for etripamil nasal spray, a calcium channel blocker that can be self-administered, found significant reductions in ED visits. Etripamil (CARDAMYST) was recently approved by the US Food and Drug Administration for the conversion of acute, symptomatic episodes of PSVT to sinus rhythm in adults.

This study is the first in recent years to characterize ED visits for PSVT. Murman et al,^16^ using a similar data source but between 1993 and 2003, estimated a rate of 50,000 ED visits annually with the ICD-9-CM PSVT code previously used in the first, second, or third diagnosis position. Our estimates reflect more current data and the currently used ICD-10-CM diagnosis code, thereby expanding our understanding of the health care burden of PSVT among prevalent patients. In 2019, there were approximately 140,000 ED visits with a PSVT diagnosis in a primary position and approximately 260,000 ED visits that included all ED visits that resulted in discharge. PSVT ED visits identified using both definitions were similar in terms of patient age, gender, and insurance coverage, as well as the proportion with AFib/AFl. Using the expanded definition, however, a larger proportion of ED visits were accompanied by observation stays, potentially reflecting the overall lower rates of admission from the ED. Given that reimbursement for ED visits where the patient is discharged does not rely on diagnosis or diagnosis position, it is possible that this higher estimate more accurately reflects ED visits for PSVT. Further, if some PSVT episodes resolve before a patient is treated in the ED, it is possible that the “true” number of ED visits for PSVT may be higher.

ED visits for PSVT increased significantly from 2016-2019 in the US, suggesting a growing burden of this cardiac arrhythmia. Model estimates of PSVT ED visits predict a continued rise in the coming years, highlighting the importance of other observed trends. Acute treatments that enable outpatient management of PSVT episodes are limited, and ED visits are burdensome and costly. Effective self-management options that reduce the need for ED visits or hospitalizations to manage PSVT episodes would provide substantial benefit to patients, providers, and payers.

## Funding and Support

Funded by Milestone Pharmaceuticals USA Inc.

## Conflict of Interest

Anita Holtz reports a relationship with Milestone Pharmaceuticals USA Inc that includes: employment and equity or stocks. David B. Bharucha reports a relationship with Milestone Pharmaceuticals USA Inc that includes: employment and equity or stocks. Sean D. Pokorney reports a relationship with Milestone Pharmaceuticals USA Inc that includes: consulting or advisory. Nihar R. Desai reports a relationship with Milestone Pharmaceuticals USA Inc that includes: consulting or advisory. Charles V. Pollack reports a relationship with Milestone Pharmaceuticals USA Inc that includes: consulting or advisory. Arjun K. Venkatesh reports a relationship with Milestone Pharmaceuticals USA Inc that includes: consulting or advisory. Naomi Sacks is an employee of BlueRidge Life Sciences, which received funding from Milestone Pharmaceuticals for this study. Xiahui Jiang is an employee of BlueRidge Life Sciences, which received funding for this study.
