# Supplementary material for: Emergency Department Visits in the United States for Paroxysmal Supraventricular Tachycardia Are Increasing Among Adults: An Analysis from the Nationwide Emergency Department Sample
Source: J Am Coll Emerg Physicians Open. 2026 Mar 3;7(2):100343. doi: 10.1016/j.acepjo.2026.100343 (PMC13055564; doi:10.1016/j.acepjo.2026.100343)
Supplement: Supplementary Tables 1-9 [file mmc1.docx]

**Supplementary Table 1.** The International Classification of Diseases, Tenth Revision, Clinical Modification (ICD-10-CM) codes for atrial fibrillation (AFib) or atrial flutter (AFl).

| **ICD-10-CMCodes** | **Description** |
| --- | --- |
| I48.0 | Paroxysmal atrial fibrillation |
| I48.1x | Persistent atrial fibrillation |
| I48.2x | Chronic atrial fibrillation |
| I48.3 | Typical atrial flutter |
| I48.4 | Atypical atrial flutter |
| I48.9x | Unspecified atrial fibrillation and atrial flutter |

**Supplementary Table 2**. ED Visits among Adults age 18+ in the United States with PSVT as Primary Diagnosis, Nationwide Emergency Department Sample, 2016-2019

|  | **2016** | **2017** | **2018** | **2019** |
| --- | --- | --- | --- | --- |
| Total ED visits | 116033535 | 118034396 | 114821688 | 117352776 |
| Number of PSVT ED visits (95% CI),  % of total ED visits^1^ | 129219 (121930, 136507), 0.111% | 132923 (125396, 140450), 0.113% | 133631 (126849, 140414), 0.116% | 139992 (132913, 147070), 0.119% |
| Rate (95% CI)^2,4^ | 5.16 (4.87, 5.45) | 5.26 (4.97, 5.56) | 5.25 (4.98, 5.51) | 5.46 (5.18, 5.73) |
| Admitted to the same hospital as ED, N (%) | 30764 (23.8%) | 31339 (23.6%) | 31809 (23.8%) | 33835 (24.2%) |
| Observation stays, N (% of PSVT ED visits) | 9840 (7.6%) | 13609 (10.2%) | 12485 (9.3%) | 14113 (10.1%) |
| Age, mean (95% CI) | 57.4  (57.1, 57.7) | 57.6  (57.3, 58.0) | 57.8  (57.5, 58.2) | 57.8  (57.4, 58.1) |
| Age group, N (%) |  |  |  |  |
| 18-64^3^ | 82387 (63.8%) | 84317 (63.4%) | 83312 (62.3%) | 87686 (62.6%) |
| …18-49 | 41353 (32.0%) | 41028 (30.9%) | 41405 (31.0%) | 43519 (31.1%) |
| …50-54 | 12869 (10.0%) | 12867 (9.7%) | 12337 (9.2%) | 12890 (9.2%) |
| …55-59 | 14278 (11.0%) | 15412 (11.6%) | 14689 (11.0%) | 15458 (11.0%) |
| ...60-64 | 13886 (10.7%) | 15010 (11.3%) | 14881 (11.1%) | 15819 (11.3%) |
| 65 and above^1^ | 46832 (36.2%) | 48606 (36.6%) | 50319 (37.7%) | 52305 (37.4%) |
| …65-69 | 13550 (10.5%) | 13990 (10.5%) | 14304 (10.7%) | 14609 (10.4%) |
| …70-74 | 10737 (8.3%) | 11269 (8.5%) | 12178 (9.1%) | 12787 (9.1%) |
| …75-79 | 8379 (6.5%) | 9050 (6.8%) | 9467 (7.1%) | 9920 (7.1%) |
| …80-84 | 6826 (5.3%) | 6645 (5.0%) | 6704 (5.0%) | 7017 (5.0%) |
| …85 and above | 7340 (5.7%) | 7653 (5.8%) | 7666 (5.7%) | 7972 (5.7%) |
| Gender, N (%) |  |  |  |  |
| Male | 51533 (39.9%) | 53160 (40.0%) | 54440 (40.7%) | 56948 (40.7%) |
| Female | 77640 (60.1%) | 79752 (60.0%) | 79191 (59.3%) | 83035 (59.3%) |
| Expected primary payer, N (%) |  |  |  |  |
| Medicare | 48826 (37.8%) | 50529 (38.0%) | 51888 (38.8%) | 53028 (37.9%) |
| Medicaid | 16438 (12.7%) | 17054 (12.8%) | 16825 (12.6%) | 17349 (12.4%) |
| Private insurance | 50521 (39.1%) | 51596 (38.8%) | 50993 (38.2%) | 55110 (39.4%) |
| Other/Unknown^5^ | 13433 (10.4%) | 13744 (10.3%) | 13925 (10.4%) | 14505 (10.4%) |
| Region of hospital, N (%) |  |  |  |  |
| Northeast | 20727 (16.0%) | 21610 (16.3%) | 23461 (17.6%) | 24120 (17.2%) |
| Midwest | 31169 (24.1%) | 30781 (23.2%) | 31229 (23.4%) | 33417 (23.9%) |
| South | 48424 (37.5%) | 50780 (38.2%) | 50301 (37.6%) | 51913 (37.1%) |
| West | 28899 (22.4%) | 29752 (22.4%) | 28641 (21.4%) | 30542 (21.8%) |

^1^ p-trend < 0.0001

^2^ p-trend < 0.01

^3^ p-trend < 0.05

^4^ Rates per 10,000 calculated using the US population counts from 2016-2019 as the denominator

^5^ Other insurance includes self-pay, no charge, missing, invalid

**Supplementary Table 3.** ED Visits among Adults age 18+ in the United States with PSVT as Primary Diagnosis without secondary AFib/AFl diagnosis, Nationwide Emergency Department Sample, 2016-2019

|  | **2016** | **2017** | **2018** | **2019** |
| --- | --- | --- | --- | --- |
| Total ED visits | 116033535 | 118034396 | 114821688 | 117352776 |
| Number of PSVT ED visits (95% CI),  % of total ED visits^2^ | 113402 (107186, 119617), 0.098% | 115762 (109339, 122185), 0.098% | 114883 (109114, 120651), 0.100% | 120377 (114389, 126364), 0.103% |
| Rate (95% CI)^3, 4^ | 4.53 (4.28, 4.78) | 4.58 (4.33, 4.84) | 4.51 (4.29, 4.74) | 4.69 (4.46, 4.93) |
| Admitted to the same hospital as ED, N (%) | 21545 (19.0%) | 21306 (18.4%) | 21168 (18.4%) | 22622 (18.8%) |
| Observation stays, N (%) | 8437 (7.4%) | 11684 (10.1%) | 10389 (9.0%) | 12032 (10.0%) |
| Age, mean (95% CI) | 55.9  (55.6, 56.3) | 56.1  (55.8, 56.5) | 56.3  (55.9, 56.6) | 56.2  (55.9, 56.5) |
| Age group, N (%) |  |  |  |  |
| 18-64^3^ | 76506 (67.5%) | 77967 (67.4%) | 76223 (66.3%) | 80173 (66.6%) |
| …18-49 | 39549 (34.9%) | 39020 (33.7%) | 39157 (34.1%) | 41103 (34.1%) |
| …50-54 | 11887 (10.5%) | 11866 (10.3%) | 11246 (9.8%) | 11758 (9.8%) |
| …55-59 | 12853 (11.3%) | 13858 (12.0%) | 13070 (11.4%) | 13810 (11.5%) |
| ...60-64 | 12217 (10.8%) | 13223 (11.4%) | 12750 (11.1%) | 13503 (11.2%) |
| 65 and above^1^ | 36896 (32.5%) | 37795 (32.6%) | 38659 (33.7%) | 40203 (33.4%) |
| …65-69 | 11545 (10.2%) | 11835 (10.2%) | 11832 (10.3%) | 12201 (10.1%) |
| …70-74 | 8672 (7.6%) | 8931 (7.7%) | 9773 (8.5%) | 10121 (8.4%) |
| …75-79 | 6380 (5.6%) | 6891 (6.0%) | 7051 (6.1%) | 7252 (6.0%) |
| …80-84 | 4968 (4.4%) | 4812 (4.2%) | 4857 (4.2%) | 5042 (4.2%) |
| …85 and above | 5330 (4.7%) | 5325 (4.6%) | 5147 (4.5%) | 5588 (4.6%) |
| Gender, N (%) |  |  |  |  |
| Male | 43779 (38.6%) | 44939 (38.8%) | 45440 (39.6%) | 47306 (39.3%) |
| Female | 69589 (61.4%) | 70812 (61.2%) | 69442 (60.4%) | 73062 (60.7%) |
| Expected primary payer, N (%) |  |  |  |  |
| Medicare | 38590 (34.0%) | 39500 (34.1%) | 40088 (34.9%) | 40804 (33.9%) |
| Medicaid | 15284 (13.5%) | 15569 (13.4%) | 15302 (13.3%) | 15711 (13.1%) |
| Private insurance | 46977 (41.4%) | 47803 (41.3%) | 46614 (40.6%) | 50473 (41.9%) |
| Other/Unknown^5^ | 12551 (11.1%) | 12891 (11.1%) | 12879 (11.2%) | 13389 (11.1%) |
| Region of hospital, N (%) |  |  |  |  |
| Northeast | 17789 (15.7%) | 18392 (15.9%) | 19728 (17.2%) | 20570 (17.1%) |
| Midwest | 27532 (24.3%) | 26783 (23.1%) | 26949 (23.5%) | 28713 (23.9%) |
| South | 42389 (37.4%) | 44209 (38.2%) | 43075 (37.5%) | 44344 (36.8%) |
| West | 25692 (22.7%) | 26378 (22.8%) | 25130 (21.9%) | 26749 (22.2%) |

^1^ p-trend < 0.0001

^2^ p-trend < 0.05

^3^ p-trend > 0.05

^4^ Rates per 10,000 calculated using the US population counts from 2016-2019 as the denominator

^5^ Other insurance includes self-pay, no charge, missing, invalid

**Supplementary Table 4.** ED Visits among Adults age 18+ in the United States with PSVT as Primary Diagnosis with secondary AFib/AFl diagnosis, Nationwide Emergency Department Sample, 2016-2019

|  | **2016** | **2017** | **2018** | **2019** |
| --- | --- | --- | --- | --- |
| Total ED visits | 116033535 | 118034396 | 114821688 | 117352776 |
| Number of PSVT ED visits (95% CI),  % of total ED visits ^1^ | 15817 (14516, 17118), 0.014% | 17161 (15828, 18495), 0.015% | 18749 (17478, 20020), 0.016% | 19615 (18255, 20975), 0.017% |
| Rate (95% CI)^1,2^ | 0.63 (0.58, 0.68) | 0.68 (0.63, 0.73) | 0.74 (0.69, 0.79) | 0.76 (0.71, 0.82) |
| Admitted to the same hospital as ED, N (%) | 9219 (58.3%) | 10033 (58.5%) | 10641 (56.8%) | 11213 (57.2%) |
| Observation stays, N (%) | 1402 (8.9%) | 1925 (11.2%) | 2097 (11.2%) | 2081 (11.0%) |
| Age, mean (95% CI) | 67.9 (67.2, 68.5) | 67.9 (67.3, 68.4) | 67.6 (66.9, 68.2) | 67.4 (66.9, 67.9) |
| Age group, N (%) |  |  |  |  |
| 18-64^1^ | 5881 (37.2%) | 6350 (37.0%) | 7089 (37.8%) | 7513 (38.3%) |
| …18-49 | 1805 (11.4%) | 2007 (11.7%) | 2248 (12.0%) | 2416 (12.3%) |
| …50-54 | 982 (6.2%) | 1001 (5.8%) | 1091 (5.8%) | 1132 (5.8%) |
| …55-59 | 1425 (9.0%) | 1554 (9.1%) | 1619 (8.6%) | 1648 (8.4%) |
| ...60-64 | 1669 (10.5%) | 1787 (10.4%) | 2131 (11.4%) | 2316 (11.8%) |
| 65 and above^1^ | 9936 (62.8%) | 10812 (63.0%) | 11660 (62.2%) | 12102 (61.7%) |
| …65-69 | 2005 (12.7%) | 2154 (12.6%) | 2472 (13.2%) | 2408 (12.3%) |
| …70-74 | 2064 (13.1%) | 2338 (13.6%) | 2404 (12.8%) | 2666 (13.6%) |
| …75-79 | 1999 (12.6%) | 2159 (12.6%) | 2416 (12.9%) | 2669 (13.6%) |
| …80-84 | 1858 (11.7%) | 1833 (10.7%) | 1848 (9.9%) | 1976 (10.1%) |
| …85 and above | 2009 (12.7%) | 2328 (13.6%) | 2520 (13.4%) | 2384 (12.2%) |
| Gender, N (%) |  |  |  |  |
| Male | 7754 (49.0%) | 8221 (47.9%) | 9000 (48.0%) | 9642 (49.2%) |
| Female | 8052 (50.9%) | 8940 (52.1%) | 9749 (52.0%) | 9973 (50.8%) |
| Expected primary payer, N (%) |  |  |  |  |
| Medicare | 10237 (64.7%) | 11029 (64.3%) | 11800 (62.9%) | 12224 (62.3%) |
| Medicaid | 1154 (7.3%) | 1485 (8.7%) | 1523 (8.1%) | 1638 (8.4%) |
| Private insurance | 3544 (22.4%) | 3793 (22.1%) | 4379 (23.4%) | 4637 (23.6%) |
| Other/Unknown^3^ | 882 (5.6%) | 854 (5.0%) | 1047 (5.6%) | 1116 (5.7%) |
| Region of hospital, N (%) |  |  |  |  |
| Northeast | 2938 (18.6%) | 3218 (18.8%) | 3732 (19.9%) | 3549 (18.1%) |
| Midwest | 3637 (23.0%) | 3998 (23.3%) | 4279 (22.8%) | 4704 (24.0%) |
| South | 6035 (38.2%) | 6571 (38.3%) | 7226 (38.5%) | 7569 (38.6%) |
| West | 3207 (20.3%) | 3374 (19.7%) | 3511 (18.7%) | 3793 (19.3%) |

^1^ p-trend < 0.0001

^2^ Rates per 10,000 calculated using the US population counts from 2016-2019 as the denominator

^3^ Other insurance includes self-pay, no charge, missing, invalid

**Supplementary Table 5**. ED Visits among Adults age 18+ in the United States with Any Position PSVT with and without Atrial Fibrillation/Atrial Flutter, Nationwide Emergency Department Sample (NEDS), 2019

|  | **Overall** | **Without AFib/AFl** | **With AFib/AFl** |
| --- | --- | --- | --- |
| Total ED visits | 117352776 | N/A | N/A |
| Number of PSVT ED visits (95% CI),  % of total ED visits | 526446 (494156, 558736), 0.449% | 3719969 (349814, 394179), 0.317% | 154450 (143612, 165287), 0.132% |
| Rate (95% CI)^1^ | 20.52  (19.26, 21.78) | 14.5  (13.64, 15.36) | 6.02 (5.60, 6.44) |
| Admitted to the same hospital as ED, N (%) | 299962 (57.0%) | 177587 (47.7%) | 122375 (79.2%) |
| Observation stays, N (%) | 45130 (19.9%) | 34720 (17.9%) | 10410 (32.5%) |
| Age, mean (95% CI) | 63.6 (63.3, 64.0) | 60.6 (60.2, 61.0) | 71.0 (70.7, 71.3) |
| Age group, N (%) |  |  |  |
| 18-64 | 246096 (46.7%) | 201942 (54.3%) | 44154 (28.6%) |
| …18-49 | 111941 (21.3%) | 99973 (26.9%) | 111941 (21.3%) |
| …50-54 | 35115 (6.7%) | 28459 (7.7%) | 35115 (6.7%) |
| …55-59 | 45608 (8.7%) | 35207 (9.5%) | 45608 (8.7%) |
| ...60-64 | 53432 (10.1%) | 38304 (10.3%) | 53432 (10.1%) |
| 65 and above | 280350 (53.3%) | 170054 (45.7%) | 110296 (71.4%) |
| …65-69 | 57176 (10.9%) | 39012 (10.5%) | 18163 (11.8%) |
| …70-74 | 59735 (11.3%) | 37580 (10.1%) | 22155 (14.3%) |
| …75-79 | 54443 (10.3%) | 32071 (8.6%) | 22371 (14.5%) |
| …80-84 | 46337 (8.8%) | 26377 (7.1%) | 19960 (12.9%) |
| …85 and above | 62660 (11.9%) | 35013 (9.4%) | 27646 (17.9%) |
| Gender, N (%) |  |  |  |
| Male | 226190 (43.0%) | 149782 (40.3%) | 76408 (49.5%) |
| Female | 300202 (57.0%) | 222176 (59.7%) | 78027 (50.5%) |
| Race/ethnicity, N (%) |  |  |  |
| White | 367393 (69.8%) | 254058 (68.3%) | 113335 (73.4%) |
| Black | 86275 (16.4%) | 63673 (17.1%) | 22602 (14.6%) |
| Hispanic | 39190 (7.4%) | 29422 (7.9%) | 9768 (6.3%) |
| Asian or Pacific Islander | 12419 (2.4%) | 9031 (2.4%) | 3388 (2.2%) |
| Native American | 1831 (0.3%) | 1376 (0.4%) | 455 (0.3%) |
| Other/Unknown^2^ | 19339 (3.7%) | 14437 (3.9%) | 4902 (3.2%) |
| Expected primary payer, N (%) |  |  |  |
| Medicare | 288961 (54.9%) | 179210 (48.2%) | 109751 (71.1%) |
| Medicaid | 63255 (12.0%) | 51114 (13.7%) | 12141 (7.9%) |
| Private insurance | 135728 (25.8%) | 109776 (29.5%) | 25952 (16.8%) |
| Other/Unknown^3^ | 38502 (7.3%) | 31897 (8.6%) | 6605 (4.3%) |
| Region of hospital, N (%) |  |  |  |
| Northeast | 92724 (17.6%) | 64486 (17.3%) | 28238 (18.3%) |
| Midwest | 134260 (25.5%) | 94068 (25.3%) | 40192 (26.0%) |
| South | 188914 (35.9%) | 134126 (36.1%) | 54787 (35.5%) |
| West | 110549 (21.0%) | 79316 (21.3%) | 31232 (20.2%) |

^1^ Rates per 10,000 calculated using the US population counts from 2016-2019 as the denominator

^2^ Other race/ethnicity includes other, missing, invalid

^3^ Other insurance includes self-pay, no charge, missing, invalid

**Supplementary Table 6**: ED Visits among Adults age 18+ in the United States with Primary PSVT Admitted plus Any Position PSVT Treated and Released, Nationwide Emergency Department Sample, 2016-2019

|  | **2016** | **2017** | **2018** | **2019** |
| --- | --- | --- | --- | --- |
| Total ED visits | 116033535 | 118034396 | 114821688 | 117352776 |
| Number of PSVT ED visits (95% CI),  % of total ED visits^1^ | 212374 (198680, 226068), 0.183% | 229549 (213787, 245310), 0.194% | 241259 (226706, 255811), 0.210% | 259916 (243277, 276554), 0.221% |
| Rate (95% CI)^1,2^ | 8.48  (7.94, 9.03) | 9.09  (8.47, 9.71) | 9.48  (8.90, 10.05) | 10.13  (9.48, 10.78) |
| Admitted to the same hospital as ED, N (%) | 30764 (14.5%) | 31339 (13.7%) | 31809 (13.2%) | 33835 (13.0%) |
| Observation stays, N (%) | 25953 (14.3%) | 37823 (19.1%) | 39833 (19.0%) | 45075 (19.9%) |
| Age, mean (95% CI) | 57.6  (57.1, 58.1) | 57.8   (57.4, 58.2) | 58.0  (57.6, 58.4) | 58.0  (57.6, 58.4) |
| Age group, N (%) |  |  |  |  |
| 18-64^1^ | 131310 (61.8%) | 141199 (61.5%) | 146154 (60.6%) | 157785 (60.7%) |
| …18-49 | 69957 (32.9%) | 74229 (32.3%) | 77675 (32.2%) | 83531 (32.1%) |
| …50-54 | 18964 (8.9%) | 20201 (8.8%) | 20320 (8.4%) | 21877 (8.4%) |
| …55-59 | 21367 (10.1%) | 23533 (10.3%) | 23897 (9.9%) | 25916 (10.0%) |
| ...60-64 | 21023 (9.9%) | 23237 (10.1%) | 24262 (10.1%) | 26461 (10.2%) |
| 65 and above^1^ | 81064 (38.2%) | 88350 (38.5%) | 95105 (39.4%) | 102130 (39.3%) |
| …65-69 | 20930 (9.9%) | 22181 (9.7%) | 24089 (10.0%) | 25237 (9.7%) |
| …70-74 | 17733 (8.3%) | 19533 (8.5%) | 21511 (8.9%) | 23399 (9.0%) |
| …75-79 | 15064 (7.1%) | 16540 (7.2%) | 17937 (7.4%) | 19343 (7.4%) |
| …80-84 | 12444 (5.9%) | 13357 (5.8%) | 14106 (5.8%) | 15411 (5.9%) |
| …85 and above | 14893 (7.0%) | 16739 (7.3%) | 17461 (7.2%) | 18740 (7.2%) |
| Gender, N (%) |  |  |  |  |
| Male | 79206 (37.3%) | 84759 (36.9%) | 90484 (37.5%) | 98623 (37.9%) |
| Female | 133098 (62.7%) | 144681 (63.0%) | 150775 (62.5%) | 161258 (62.0%) |
| Expected primary payer, N (%) |  |  |  |  |
| Medicare | 85750 (40.4%) | 93488 (40.7%) | 100421 (41.6%) | 106844 (41.1%) |
| Medicaid | 29014 (13.7%) | 32554 (14.2%) | 33176 (13.8%) | 36187 (13.9%) |
| Private insurance | 76761 (36.1%) | 80973 (35.3%) | 84425 (35.0%) | 91900 (35.4%) |
| Other/Unknown^3^ | 20848 (9.8%) | 22534 (9.8%) | 23236 (9.6%) | 24985 (9.6%) |
| Region of hospital, N (%) |  |  |  |  |
| Northeast | 30042 (14.1%) | 32242 (14.0%) | 36518 (15.1%) | 38123 (14.7%) |
| Midwest | 54844 (25.8%) | 57097 (24.9%) | 59488 (24.7%) | 69341 (26.7%) |
| South | 79511 (37.4%) | 88764 (38.7%) | 92028 (38.1%) | 94332 (36.3%) |
| West | 47977 (22.6%) | 51446 (22.4%) | 53225 (22.1%) | 58120 (22.4%) |

^1^ p-trend < 0.0001

^2^ Rates per 10,000 calculated using the US population counts from 2016-2019 as the denominator

^3^ Other insurance includes self-pay, no charge, missing, invalid

**Supplementary Table 7**: ED Visits among Adults age 18+ in the United States with Primary PSVT Admitted plus Any Position PSVT Treated and Released without any position AFib/AFl diagnosis, Nationwide Emergency Department Sample, 2016-2019

|  | **2016** | **2017** | **2018** | **2019** |
| --- | --- | --- | --- | --- |
| Total ED visits | 116033535 | 118034396 | 114821688 | 117352776 |
| Number of PSVT ED visits (95% CI),  % of total ED visits^1^ | 182514 (171228, 193800), 0.157% | 194846 (181898, 207793), 0.165% | 201347 (189637, 213056), (0.175% | 216724 (203158, 230291), 0.185% |
| Rate (95% CI)^1,2^ | 7.29  (6.84, 7.74) | 7.72  (7.20, 8.23) | 7.91  (7.45, 8.37) | 8.45  (7.92, 8.98) |
| Admitted to the same hospital as ED, N (%) | 21545 (11.8%) | 21306 (10.9%) | 21168 (10.5%) | 22622 (10.4%) |
| Observation stays, N (%) | 20681 (12.8%) | 30297 (17.5%) | 30749 (17.1%) | 34683 (17.9%) |
| Age, mean (95% CI) | 55.9   (55.5, 56.4) | 56.0  (55.6, 56.4) | 56.0  (55.6, 56.4) | 56.1  (55.7, 56.5) |
| Age group, N (%) |  |  |  |  |
| 18-64^1^ | 120446 (66.0%) | 128366 (65.9%) | 131488 (65.3%) | 141458 (65.3%) |
| …18-49 | 66169 (36.3%) | 69755 (35.8%) | 72365 (35.9%) | 77816 (35.9%) |
| …50-54 | 17251 (9.5%) | 18210 (9.3%) | 18127 (9.0%) | 19376 (8.9%) |
| …55-59 | 18892 (10.4%) | 20547 (10.5%) | 20770 (10.3%) | 22508 (10.4%) |
| ...60-64 | 18134 (9.9%) | 19854 (10.2%) | 20227 (10.0%) | 21759 (10.0%) |
| 65 and above^1^ | 62068 (34.0%) | 66480 (34.1%) | 69859 (34.7%) | 75266 (34.7%) |
| …65-69 | 17275 (9.5%) | 18205 (9.3%) | 19199 (9.5%) | 20248 (9.3%) |
| …70-74 | 13949 (7.6%) | 14968 (7.7%) | 16400 (8.1%) | 17829 (8.2%) |
| …75-79 | 11091 (6.1%) | 12323 (6.3%) | 12877 (6.4%) | 13672 (6.3%) |
| …80-84 | 9003 (4.9%) | 9406 (4.8%) | 9804 (4.9%) | 10655 (4.9%) |
| …85 and above | 10749 (5.9%) | 11579 (5.9%) | 11580 (5.8%) | 12862 (5.9%) |
| Gender, N (%) |  |  |  |  |
| Male | 65458 (35.9%) | 68950 (35.4%) | 72400 (36.0%) | 79017 (36.5%) |
| Female | 117002 (64.1%) | 125793 (64.6%) | 128947 (64.0%) | 137673 (63.5%) |
| Expected primary payer, N (%) |  |  |  |  |
| Medicare | 66482 (36.4%) | 71193 (36.5%) | 74773 (37.1%) | 79913 (36.9%) |
| Medicaid | 26869 (14.7%) | 29604 (15.2%) | 29907 (14.9%) | 32472 (15.0%) |
| Private insurance | 69888 (38.3%) | 73339 (37.6%) | 75398 (37.4%) | 81615 (37.7%) |
| Other/Unknown^3^ | 19275 (10.6%) | 20709 (10.6%) | 21269 (10.6%) | 22724 (10.5%) |
| Region of hospital, N (%) |  |  |  |  |
| Northeast | 25905 (14.2%) | 27212 (14.0%) | 30675 (15.2%) | 32450 (15.0%) |
| Midwest | 47014 (25.8%) | 48121 (24.7%) | 49075 (24.4%) | 57072 (26.3%) |
| South | 68514 (37.5%) | 75650 (38.8%) | 76969 (38.2%) | 78694 (36.3%) |
| West | 41081 (22.5%) | 43862 (22.5%) | 44628 (22.2%) | 48509 (22.4%) |

^1^ p-trend < 0.0001

^2^ Rates per 10,000 calculated using the US population counts from 2016-2019 as the denominator

^3^ Other insurance includes self-pay, no charge, missing, invalid

**Supplementary Table 8**: ED Visits among Adults age 18+ in the United States with Primary PSVT Admitted plus Any Position PSVT Treated and Released with any position AFib/AFl diagnosis, Nationwide Emergency Department Sample, 2016-2019

|  | **2016** | **2017** | **2018** | **2019** |
| --- | --- | --- | --- | --- |
| Total ED visits | 116033535 | 118034396 | 114821688 | 117352776 |
| Number of PSVT ED visits (95% CI),  % of total ED visits^1^ | 29860 (27203, 32516), 0.026% | 34703 (31617, 37789), 0.029% | 39912 (36794, 43031), 0.035% | 43191 (39822, 46560), 0.037% |
| Rate (95% CI)^1,2^ | 1.19  (1.09, 1.30) | 1.37  (1.25, 1.50) | 1.57  (1.45, 1.69) | 1.68  (1.55, 1.81) |
| Admitted to the same hospital as ED, N (%) | 9219 (30.9%) | 10033 (28.9%) | 10641 (26.7%) | 11213 (26.0%) |
| Observation stays, N (%) | 5272 (25.5%) | 7526 (30.5%) | 9083 (31.0%) | 10392 (32.5%) |
| Age, mean (95% CI) | 67.9   (67.3, 68.5) | 67.9  (67.3, 68.4) | 67.8   (67.3, 68.3) | 67.6   (67.1, 68.1) |
| Age group, N (%) |  |  |  |  |
| 18-64^1^ | 10864 (36.4%) | 12833 (37.0%) | 14666 (36.7%) | 16327 (37.8%) |
| …18-49 | 3787 (12.7%) | 4474 (12.9%) | 5311 (13.3%) | 5715 (13.2%) |
| …50-54 | 1713 (5.7%) | 1991 (5.7%) | 2193 (5.5%) | 2501 (5.8%) |
| …55-59 | 2475 (8.3%) | 2985 (8.6%) | 3127 (7.8%) | 3408 (7.9%) |
| ...60-64 | 2888 (9.7%) | 3382 (9.7%) | 4035 (10.1%) | 4702 (10.9%) |
| 65 and above^1^ | 18996 (63.6%) | 21870 (63.0%) | 25246 (63.3%) | 26864 (62.2%) |
| …65-69 | 3655 (12.2%) | 3976 (11.5%) | 4891 (12.3%) | 4990 (11.6%) |
| …70-74 | 3783 (12.7%) | 4565 (13.2%) | 5111 (12.8%) | 5571 (12.9%) |
| …75-79 | 3973 (13.3%) | 4217 (12.2%) | 5061 (12.7%) | 5671 (13.1%) |
| …80-84 | 3441 (11.5%) | 3951 (11.4%) | 4302 (10.8%) | 4755 (11.0%) |
| …85 and above | 4144 (13.9%) | 5160 (14.9%) | 5881 (14.7%) | 5878 (13.6%) |
| Gender, N (%) |  |  |  |  |
| Male | 13748 (46.0%) | 15809 (45.6%) | 18084 (45.3%) | 19606 (45.4%) |
| Female | 16097 (53.9%) | 18888 (54.4%) | 21828 (54.7%) | 23585 (54.6%) |
| Expected primary payer, N (%) |  |  |  |  |
| Medicare | 19269 (64.5%) | 22295 (64.2%) | 25648 (64.3%) | 26931 (62.4%) |
| Medicaid | 2145 (7.2%) | 2949 (8.5%) | 3269 (8.2%) | 3715 (8.6%) |
| Private insurance | 6872 (23.0%) | 7634 (22.0%) | 9028 (22.6%) | 10285 (23.8%) |
| Other/Unknown^3^ | 1573 (5.3%) | 1825 (5.3%) | 1968 (4.9%) | 2261 (5.2%) |
| Region of hospital, N (%) |  |  |  |  |
| Northeast | 4136 (13.9%) | 5029 (14.5%) | 5843 (14.6%) | 5673 (13.1%) |
| Midwest | 7830 (26.2%) | 8976 (25.9%) | 10413 (26.1%) | 12270 (28.4%) |
| South | 10998 (36.8%) | 13114 (37.8%) | 15059 (37.7%) | 15637 (36.2%) |
| West | 6896 (23.1%) | 7584 (21.9%) | 8597 (21.5%) | 9611 (22.3%) |

^1^ p-trend < 0.0001

^2^ Rates per 10,000 calculated using the US population counts from 2016-2019 as the denominator

^3^ Other insurance includes self-pay, no charge, missing, invalid

**Supplementary Table 9**. Projected Estimates of ED visits among Adults age 18+ in the United States with PSVT in the Primary Position, and Primary PSVT Admitted plus Any Position PSVT Treated and Released Nationwide Emergency Department Sample (NEDS), 2016-2030

| Year | US Age 18+ population ^a^ | Estimated ED visits with Primary PSVT | Estimated ED visits with Primary PSVT Admitted plus Any Position PSVT Treated and Released |
| --- | --- | --- | --- |
| 2016 | 250295904 | 128984 | 213050 |
| 2017 | 252529365 | 132292 | 227633 |
| 2018 | 254623967 | 135600 | 243063 |
| 2019 | 256550335 | 138890 | 259352 |
| 2020 | 258095320 | 142042 | 276308 |
| 2021 | 259219518 | 145025 | 293885 |
| 2022 | 260837000 | 148349 | 313166 |
| 2023 | 262916434 | 152010 | 334287 |
| 2024 | 264995869 | 155752 | 356811 |
| 2025 | 267081000 | 159579 | 380836 |
| 2026 | 268861025 | 163305 | 405994 |
| 2027 | 270641049 | 167111 | 432794 |
| 2028 | 272421074 | 170998 | 461344 |
| 2029 | 274205975 | 174971 | 491765 |
| 2030 | 275986000 | 179026 | 524160 |

^a^ 2016-2021 US population is from SEER*Stat; 2022-2030 US population is interpolated using 2023 National Population Projections Tables.
